# Electricity, water, and natural gas consumption of a residential house in Canada from 2012 to 2014

**DOI:** 10.1038/sdata.2016.37

**Published:** 2016-06-07

**Authors:** Stephen Makonin, Bradley Ellert, Ivan V. Bajić, Fred Popowich

**Affiliations:** 1 Engineering Science, Simon Fraser University, 8888 University Drive, Burnaby, British Columbia, Canada V5A 1S6; 2 Computing Science, Simon Fraser University, 8888 University Drive, Burnaby, British Columbia, Canada V5A 1S6

**Keywords:** Electrical and electronic engineering, Sustainability, Scientific data, Energy modelling, Energy

## Abstract

With the cost of consuming resources increasing (both economically and ecologically), homeowners need to find ways to curb consumption. The Almanac of Minutely Power dataset Version 2 (AMPds2) has been released to help computational sustainability researchers, power and energy engineers, building scientists and technologists, utility companies, and eco-feedback researchers test their models, systems, algorithms, or prototypes on real house data. In the vast majority of cases, real-world datasets lead to more accurate models and algorithms. AMPds2 is the first dataset to capture all three main types of consumption (electricity, water, and natural gas) over a long period of time (2 years) and provide 11 measurement characteristics for electricity. No other such datasets from Canada exist. Each meter has 730 days of captured data. We also include environmental and utility billing data for cost analysis. AMPds2 data has been pre-cleaned to provide for consistent and comparable accuracy results amongst different researchers and machine learning algorithms.

## Background & Summary

Currently, much of the world is focused on reducing electricity consumption; our increase in consumption is neither economically nor environmentally sustainable. Additionally, there is a growing consensus that environmental and economical sustainability are inextricably linked^1^. As the cost of power rises, we must find technological solutions that help reduce and optimize energy use^2,3^. Residential homes contribute about 34% to the total power consumption in the USA and their consumption is projected to increase to 39% by 2030 (ref. 4). One way to help homeowners and occupants reduce their consumption is to monitor and present how much power their appliances are using through an effective eco-feedback device or display mechanism^5,6^.

The Almanac of Minutely Power dataset (AMPds) was initially released in 2013 with one year of meter data without environmental and utility billing data^7^ (Data Citation 1). The first release also contained some data integrity issues: missing readings and a counter reset that happened with the water meters. With this second release (AMPds2) we increased the monitoring length to two years (730 days of captured data per meter). The integrity problems that existed in the first release have been corrected. We have added historical climate data, two years of hourly weather data, and two years of utility billing data.

AMPds has been used (See Google Scholar: https://scholar.google.ca/scholar?cites=9977726888743581483) and can be used in research that looks at: non-intrusive load monitoring (NILM, a.k.a. load disaggregation)^8^, energy use behaviour, eco-feedback and eco-visualizations^6,9^, application and verification of theoretical algorithms/models, appliance studies, demand forecasting, smart home frameworks, grid distribution analysis, time-series data analysis, energy efficiency studies, occupancy detection, energy polity and socio-economic frameworks^10^, and advanced metering infrastructure (AMI) analytics. Testing the accuracy performance with real-world datasets is crucial in these fields of research. Synthesized data does not realistically represent an actual dataset as ‘a real-world dataset would normally have certain complexity that is harder predict and in many cases can be very difficult to deal with’ [ref. 11, p.114].

There are indeed other datasets that exist from the USA^12–16^, Europe^17–21^, and Asia^22^. However, AMPds2 is unique for a number of different reasons. AMPds2 is the only dataset from Canada. It is the only dataset to include all three main types of consumption: electricity, water, and natural gas. Data is captured over a long period of time (two years) and is presented with minor amounts of missing data algorithmically filled in to maintain a continuous frequency of readings (once per minute). For electricity data we provide 11 measurement characteristics for each meter: voltage, current, frequency, displacement power factor, apparent power factor, real power, real energy, reactive power, reactive energy, apparent power, and apparent energy.

AMPds2 data has been cleaned to provide for consistent and comparable accuracy results amongst different researchers and machine learning algorithms. Other datasets (e.g., REDD^14^) leave the onus of data cleaning on each researcher. This means that the same dataset can be cleaned very differently. This results in an inability to reproduce and compare algorithms published.

## Methods

### Residential house characteristics

**Figure 1:**
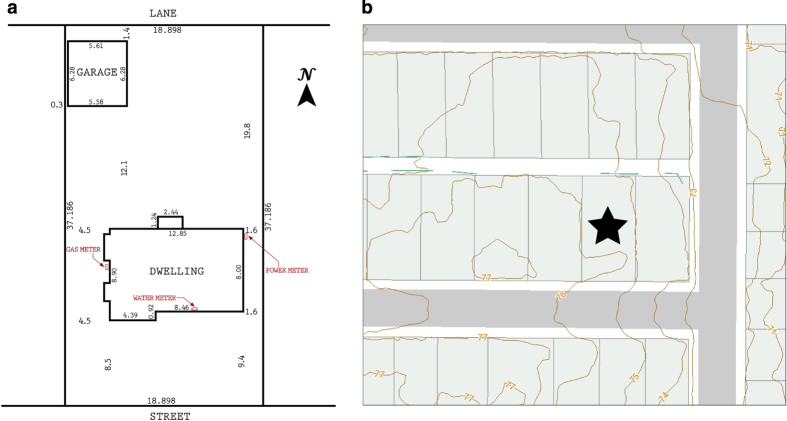
Test House Property. Surrounding area of house: (**a**) property survey and (**b**) location in surrounding block. Yellow lines show 1 m elevation contours.

Our data was collected from a house built in 1955 in the Greater Vancouver metropolitan area in British Columbia (Canada), which underwent major renovations in 2005 and 2006, receiving a Canadian Government EnerGuide^23^ rating of 82% (from 61%). The house is located in Burnaby, the municipality east of Vancouver. Elevation-wise, the house is 80 m above sea level and the front of the house faces south. The house has one level above grade and a basement making up a total of 2,140 ft^2^ (199 m^2^) of living space (1,070 ft^2^ or 99.5 m^2^ per floor). The main floor ceiling height is 8 ft (2.44 m) and the basement ceiling height is 7 ft (2.13 m). Within the house is a rental unit that takes up approximately half the basement (603 ft^2^ or 56 m^2^ of living space). The detached garage is approximately 161 ft^2^ (15 m^2^) and the overhead door faces the back alleyway (see Fig. 1).

The house has the original wood-frame construction. In 2006, all existing exterior wall stucco was removed. Proper vent covering was installed under the eaves and the exterior walls were re-stuccoed with a light green ‘California’ finish. The previous stucco finished was removed. The house has an black asphalt shingle roof that was replaced in 2007. The new asphalt shingles are light brown. When the stucco and roof were replaced, $\frac{1}{4}$-inch plywood was nailed to the existing shiplap boarding.

Originally, the above grade walls were insulated with batt insulation evaluated at R7 and the roof was insulated with blown-in insulation evaluated at R19. After renovations, R24 batt insulation was added on top of the existing ceiling insulation. The main floor wall insulation was not improved. For the basement, R24 was added to the ceiling and above grade walls. Below grade walls had R9 extruded polystyrene rigid insulation affixed to the concrete walls. The basement floor was upgraded to have DRIcore sub-flooring (see Manual_dricore.pdf, Data Citation 2) which is rated at R1.7.

Windows are double-pane low-e glass and were replaced in 2005 (see Table 1). All doors are insulated core metal and were replaced in 2005. The basement walls are approximately 25.4 cm thick with the South basement wall (front of house) almost completely below grade, while the North wall (back of house) is about 1 m below grade. The house has three full bathrooms (tub with shower, toilet, and sink) and a master bedroom ensuite (toilet and sink). Two of the bathrooms are in the basement; one is in the rec room, and the other is in the rental suite. Faucets and showerheads are restricted to a maximum flow of 9.5 l/min (2.6 GPM). All toilets have 6 l tanks and are dual-flush.

### Occupancy elaboration

The main house has a family of three persons: a male and a female adult in their late 30 s and a daughter between the age of 5 and 6. The male adult is a full-time student at a local university, the female adult is self-employed, and the child attends full-time elementary school. A rental suite houses one male occupant in his early 20 s with full-time employment.

### HVAC system elaboration

Our test house has a dual-fuel HVAC system where a heat pump is used alongside a forced air gas furnace. The heat pump cools the house in summer and heats the house in winter. The gas furnace is used, but only when it is too cold outside for the heat pump to operate effectively. When the outside ambient temperature is 2 °C or lower, the HVAC system changes over from electric heating (heat pump) to natural gas heating using the furnace. At low temperatures, heat pumps are not efficient for heating and can strain the compressor.

During data collection, the HVAC thermostat was set to a constant heating set-point of 21 °C and the cooling set-point ranged within 24–26 °C. The HVAC furnace fan was set to constantly run 24-hours to circulate the air. The furnace is 2-stage with a variable-speed fan and is rated as 93% efficient. The heat pump has a 2-stage compressor and is rated at 17 SEER. It is the central unit for air conditioning; there are no other air conditioning units in the house (besides the windows).

### Data collection

**Figure 2:**
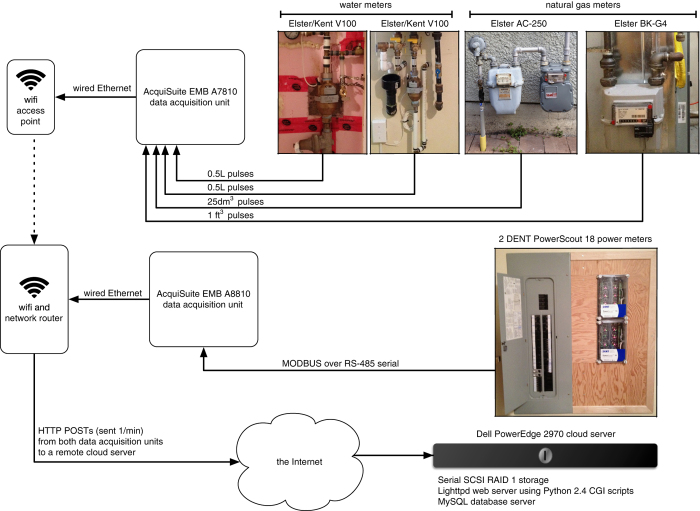
Block Diagram of Data Collection System. Electricity, water, and natural gas were monitored using industrial meters. Data was collected using industrial data acquisition units and stored offsite on a database server.

Our main concern when designing the data collection system for AMPds2 was integrity and accuracy. For these reasons we chose to use industry-standard equipment for monitoring and acquisition. Data was stored off-site on a database server that was hosted at a co-location facility with proper power backup and network connection redundancy. Figure 2 depicts the setup of our data collection system. Table 2 summarizes the specifications of the metering equipment used, including the accuracy standards each meter adheres to.

After two years of collection, only 2,029 electricity readings and 437 water and natural gas readings were missing from a total of 1,051,200 readings for each resource (discussed in more detail below for each resource). The missing readings were algorithmically created during the data cleaning process which is discussed in detail in the Dataset File Preparation Subsection^24^.

### Electricity supply & metering

BC Hydro is the provincial utility that provides electricity to the house via a 240 V, 200A service. As with all Canadian homes, two 120 V lines enter the house—leg 1 (L1) and leg 2 (L2) of the same phase. There are pole transformers that convert the single phase into two legs. Each transformer services about five homes.

Electricity measurements were taken by two DENT PowerScout 18 units metering 24 loads at the electrical circuit breaker panel. Only 21 loads were kept. The three loads that were removed were: the gas stovetop plug breaker, the microwave plug breaker, and a randomly chosen lighting breaker because no activity was recorded. All current and all current-based measurements were recorded as zero. The gas stovetop only used electricity to ignite the gas burners. The microwave had never been used and was removed at one point. The lighting breaker that was chosen was for a backyard outside light that was never used—the bulb was burned out and not replaced.

Measurements were read over a RS-485/Modbus communication link by a Obvius AcquiSuite EMB A8810 data acquisition unit. During the data cleaning process for electricity, we found and corrected 55 readings where 1 of 21 meters had missing measurements and 2,029 readings where more than one of 21 meters had missing measurements (see Dataset File Preparation Subsection for more details).

#### Water supply & metering

Burnabys water distribution system is fed by four water pump stations, four water reservoirs, and twenty-one pressure reducing stations to control and regulate water pressure. Water pressure is produced by gravity from the higher elevation water reservoirs that Metro Vancouver manages.

Water service is via a $\frac{3}{4}$-inch pipe at a pressure between 108–118 psi (744.6–813.6 kPa) [reported by Engineering Department]. A pressure regulator is used (see specifications in Manual_WilkinsModel70.pdf, Data Citation 2) to maintain water pressure in the house at 60 psi (413.7 kPa).

Water measurements were taken by 2 Elster/Kent V100 water meters, which also send pulses to a data acquisition unit. These water meters are volumetric cold water meters that measure water with a rotary piston. Before July 14, 2012 (timestamp 1342287780) the water main was metered by a DLJ 75C meter and hot water was metered by an Elster S130 meter. These meters pulse once per gallon which was too coarse of a measurement for the amount of water being consumed by the houses occupants. This was the reason for replacing these meters with ones that pulse more frequently. See Table 2 for details on these water meters (e.g., standards compliance and accuracy data).

Pulse data was collected using an Obvius AcquiSuite EMB A7810. To note, the Obvius AcquiSuite units have a per-minute sampling limitation. It is not possible to capture data at a faster rate, which is an acceptable cost for reliability. During the data cleaning process for water, we found and corrected 437 readings that were missing from both water meters.

Dishwasher water (DWW) consumption data was annotated by hand^25,26^. Having the electricity consumption data and details in the appliance manual about how the dishwater used water made this task relatively easy. This is further discussed in the Technical Validation section.

### Natural gas supply & metering

Natural gas is supplied to the house by FortisBC at a pressure of 1.75 kPa and is composed of methane, ethane, propane, and butane. FortisBC uses the Higher Heating Value (HHV) as the conversion factor when converting from gas volume to energy used in gigajoules (GJ). HHV is the total heat obtained from combustion. The heating value of the gas is measured daily by FortisBC (see file NaturalGas_HeatValues, Data Citation 2). For the Lower Mainland (Zone 24) the measurement *energy desity values* are in GJ/10^3^m^3^. FortisBC assumes a temperature of 15 °C and a pressure of 101.325 kPa for conversion of gas values into energy values.

Natural gas measurements were taken by an Elster AC250 gas meter and a Elster BK-G4 gas meter; both send pulses to a data acquisition unit. These natural gas meters are diaphragm meters. See Table 2 for details on meter standards compliance and accuracy. Pulse data was collected using an Obvius AcquiSuite EMB A7810. During the data cleaning process for gas, we found and corrected 437 readings that were missing from both gas meters.

### Environmental & weather records

Hourly weather data was downloaded from the Environment Canadas Weather Office which has a weather station at YVR (Vancouver International Airport) located at latitude of 49.20, longitude of −123.18, and elevation of 4.30 m. Our test house is approximately 18 km from YVR with an elevation difference of approximately 75 m. YVR is located next to the water which might account for slight differences in outdoor temperature between the two locations. There is no precise method to determine this difference. Anecdotally we have seen up to ±2 °C. Date and times listed within this file are in Local Standard Time (LST). Add 1 h to adjust for Daylight Saving Time when it is observed. The *Data Quality* column (and other columns) may contain M (missing), E (estimated), NA (not available), or ** (Partner data that is not subject to review by the National Climate Archives).

Historical climate normals data (from 1981 to 2010) was downloaded from the Environment Canadas Weather Office which had a weather station at Burnaby Capitol Hill (latitude of 49.17, longitude of −122.59, and elevation of 182.9 m). This weather station was closer to our test house but closed down in 2010. Precipitation data about rainfall and snowfall is included.

### Utility bills & invoice records

The billing data for all three forms of consumption was created from the values that exist on the included redacted utility billing statements. We were able to download 50% of the billing data from our account on the utility's website. The remaining data was manually entered in. All billing data was human verified for accuracy from each billing statement. Data entered by hand was rechecked for accuracy after the values of each bill were recorded.

### Code availability

Code used to store data collected via the data acquisition units to the database server can be download from the online code repository GitHub^24^. The scripts used to convert the database tables to the final dataset files can be downloaded from the same online code repository (see the Technical Validation section).

## Data Records

AMPds2 is publicly available for download from Harvard Dataverse (Data Citation 2) in many different formats including: the original CSV, tab-delimited, and RData format. Table 3 lists a description of each file that is part of AMPds2. File names describe the contents by listing the type of data and the meter ID separated by an underscore. There are four types of data: Electricity, Water, NaturalGas, and Climate. For example, Electricity_CDE.csv would be electricity data from the clothes dryer (CDE) meter, NaturalGas_Billing.csv would be natural gas billing data. Refer to Table 3 for a description of all files included in the AMPds2 dataset. Refer to Table 4 (available online only) for a description of meter IDs and datafile column names.

Each row in each of the dataset files represents a single meter reading once every minute with an associated unix timestamp. Each reading contains all the measurements and calculations provided by the meter. Refer to Table 4 (available online only) for specific information on each measurement provided. In the case of pulse metering, the data acquisition unit calculated the three measurements (counter, avg_rate, inst_rate) as pulses were received from each meter.

This integer timestamp is the amount of seconds since 1970-01-01 12:00:00am (UTC). Because each reading is one minute apart the timestamp number increases by 60 every reading. The two data acquisition units use the Network Time Protocol (NTP) for clock synchronization. There were records where the timestamp was off by ±10 s. In these cases our data cleaning script^24^ corrected the timestamp to have zero-seconds. This slight variation in time was caused by having to download the readings of 24 loads over a limiting fixed baud rate (of 9600 bps) used by the DENT meters.

**Figure 3:**
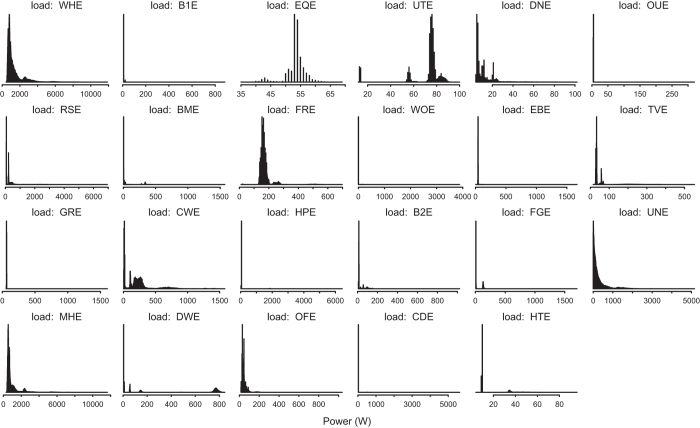
Load Profiles. Histograms showing the load profile for each of the electricity meters.

**Figure 4:**
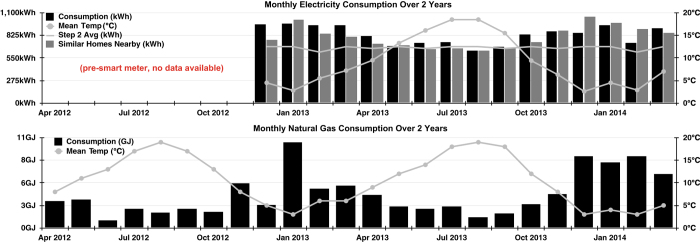
Monthly Consumption Chart. Monthly consumption charts of both electricity (top) and natural gas (bottom) data received from each utility company.

**Figure 5:**
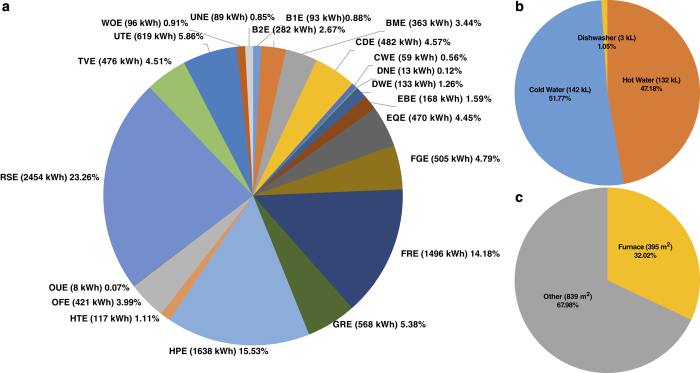
Yearly Load Consumption Breakdown. The amount of yearly consumption for metered appliances averaged over the two year period of (**a**) electricity (total of 10,550 kWh), (**b**) water (total of 277 kl), and (**c**) natural gas (total of 1235 m^3^).

Table 4 (available online only) describes the column names found within each file. No one file will contain all the column names listed. Figure 3, Fig. 4, and Fig. 5 give some insight as to how the house consumed resources over the two years. Additionally, Table 5 (available online only) gives detailed information about each of the major appliances that consumed resources in our test house.

Climate data files are kept in the original format provided by Environment Canada. Each row in Electricity_Billing.csv and NaturalGas_Billing.csv will match a utility billing statement found in Electricity_Statements.csv and NaturalGas_Statements.csv, respectively. Statements are not available for Water_Billing.csv data.

### One-time events & oddities

On May 4, 2012 at 10:34am local time (timestamp 1336152840) the houses existing electro-mechanical meter was replaced with a digital ‘smart’ meter. This explains why all electricity reading were recorded as zero.

On July 14, 2012 between 10:43am and 5:03pm local time the houses water supply was disconnected to perform a repair. The instantaneous hot water unit has internal leaking due to micro-imperfections in the copper pipe. During this time the existing water meters (which pulsed at a per gallon rate) were replaced with water meters that pulse per 0.5 l.

During the period of data collection, the main house family when on holidays/travel during the following periods: May 1–7, 2012; June 9–18, 2012; and, July 31—August 8, 2013. Consumption during these times should be near zero. The rental occupant was not tracked, in terms of taking holidays/travel.

## Technical Validation

The meters and data acquisition equipment were manufactured by well known companies that produce meters for industrial and residential installations around the world. Meter calibration was done by the meter manufacture before shipping at the factory. The calibration process is proprietary and we were not privy to the process.

### Dataset file preparation

Scripts were created to export the data from the database to final comma separated values (CSV) files. During this process we checked the integrity of the data. If readings were missing they were algorithmically added^24^. To note these additions, a plus sign was added to the beginning of each timestamp, which does not affect the programatic conversion from a string to an integer. Our data cleaning scripts (make_AMPds2_power.py and make_AMPds2_pulse.py) work as follows:

1. From MySQL export data into CSV files, 1 raw file/meter

2. Execute./make_AMPds2_power.py or make_AMPds2_pulse.py.

3. Load all raw data CSV files into memory.

4. Create empty records that will store clean data.

5. For each meter and each CSV row.

6. Zero out the seconds in the timestamp.

7. Convert the timestamp to a record index i.

8. If this record at index i is empty then.

9. Convert each measurement to the proper data type.

10. Add the measurements to this record.

11. For each record in the CDE meter.

12. Fix record by removing phantom 0.4A and 27–30VA.

13. For timestamp and each meter.

14. Use equations (1) and (2) so WHE >=MHE+RSE+GRE.

15. If the previous records was missing data then.

16. Fill in the missing measurement data.

17. Event distribute the accumulation for Pt, Qt, St.

18. Save clean records for each meter

### Soft-meter calculations

**Figure 6:**
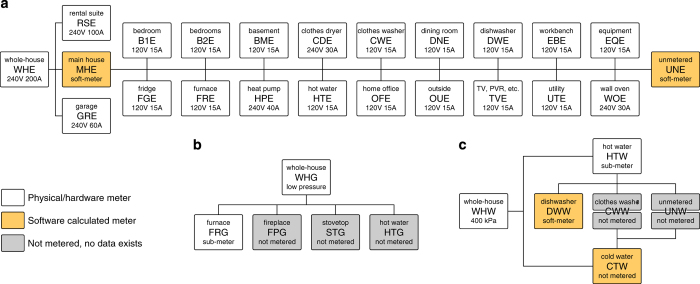
Metering Bus Diagrams. Bus diagrams of how each of the (**a**) electricity, (**b**) natural gas, and (**c**) water meters are connected in relation to each other. For natural gas and water, we show what other appliances/loads exist that consume each resource. This is not done for electricity because there are too many unmetered loads.

Soft-meter data was calculated during this process. Figure 6 shows how each meter is related to each other and which meters are soft-meters. The main house electricity soft-meter (MHE) is calculated by the formula

(1)MHE=WHE−(RSE+GRE).

The unmetered electricity soft-meter (UNE) is calculated by the formula

(2)UNE=MHE−(B1E+B2E+BME+CDE+CWE+DNE+DWE+EBE+EQE+FGE+FRE+HPE+HTE+OFE+OUE+TVE+UTE+WOE).

To calculate cold water consumption use the formula

(3)CTW=WHW−HTW.

The calculation of cold water will work over longer periods of time (say one day). Equation (3) will not work over shorter periods of time, because the water meters are pulsing at coarse values of 0.5 l where the time between pulses may cross over multiple minutes where small amounts of water are used.

The dishwasher water soft-meter (DWW) was manually annotated as discussed previously^25,26^. DWW consumption followed a very specific pattern of 3 l spurts of water correlating to patterns in the electrical data. In most cases, this was the only water being consumed in the house, making the annotation as simple as copying these readings. When there was simultaneous water use, usually the signal could easily be visually decomposed and/or a nearby reading could be used to infer the proper labelling. There were very few cases where an arbitrary choice between two equally likely labellings had to be made.

### Measurement uncertainty between main & sub-meters

The DENT power meter used is considered revenue class (Class 0.5) which has a very high accuracy, typically better than 1% (<0.5% typical). This meter accuracy classes are governed by two standards organizations: ANSI C12.20 for North America and IEC 62053 elsewhere (see Table 2).

For this class of meter the absolute error is limited to the 0.5% of the full scale reading. Usually, however, the error is somewhat proportional to the reading, with higher readings subject to larger absolute error than lower-valued readings. To make a simple model, we could consider each meter to add a Gaussian error to the true value, with variance proportional to the true value. Each individual meter adds such Gaussian noise. So the variance of the sum of such readings is the sum of individual variances (i.e., proportional to the sum of true values). According to the same model, the main meter makes a Gaussian error with variance proportional to the whole-house power usage, which is the sum of true power values in each individual meter. Hence, the error in the main meter has the same variance as the sum of the readings of individual meters. This is due to the fact that all meters are Class 0.5, so we expect they would have the same constant of proportionality for the variance. If the main meter had a higher class (better rating than 0.5%), then it would produce less uncertainty than the sum of individual meters.

### Dataset cleaning

For the electricity data, an additional step was performed. We checked that the whole-house reading was never less than the summation of all sub-meters. If it was then the whole-house reading was changed to be equal to the summation. This can happen because not all meters can be read simultaneously. Each DENT PowerScout 18 meter has 6 three-phase sub-meters (labelled A through F) which can be configured to be 18 single-phase sub-meters. The storage registers within each of the 6 three-phase sub-meters is updated once per second with new measurements. Previously, we discussed the issue of timestamp synchronization and that timestamps between sub-meters could be off by ±10 s due to the fact that the meters have a limiting fixed baud rate of 9600 bsp. This slight variation in reading time is the cause of having whole-house readings less than the summation of all sub-meters. Suppose, for example, the electricity mains are metered by sub-meter A and the heat pump is metered by sub-meter F. The data acquisition unit would download the measurement data from sub-meter A, then B, and so on, finally to F—taking a total of 10 s to do. If the heat pump was to turn ON within that 10 s window, then the readings from sub-meter A would not reflect the more recent event that would be reflected in sub-meter F—the heat pump turning ON.

The second factor that can contribute to this summation has to do with rounding. Although the meter is quite precise, the measurement values stored in the memory registers are rounded to the nearest whole number for some measurements and tenths of a whole number for other measurements. When we sum up these rounded numbers, they can exceed the whole-house reading. No changes to the whole-house reading were performed if the opposite was true. This is because there were many unmetered loads in the house that could be running at any given time.

We found an additional problem that affected the metering of the clothes dryer (CDE) PowerScout 18 Unit 1 Meter E. L3 (line 3, for 3-phase loads) was not used but the meter was recording a phantom load for 04.A and between 27–30VA. We verified with a multimeter that this should not be the case. There is an additional step to remove the phantom load measurements from the CDE datafile. For details, refer to the make_AMPds2_power.py script^24^.

For the water data, 14 discrepancies were found between the counter and avg_rate. In all cases, the counter should be a cumulative sum of the avg_rate. Of the few times when this was not the case, usually (9 out of 14 times) it was because a pulse failed to be recorded in the avg_rate column. In a few cases (4 out of 14 times), the avg_rate was not a multiple of the pulse size. For both of these types of error, the avg_rate was simply overwritten with the true value derived from the change in the counter. The remaining occurrence (1 out of 14 times) was an accuracy error of 0.001 in the counter. This and all following counter values were adjusted to fix this. For details, refer to the make_AMPds2_pulse.py script^24^.

## Additional Information

**How to cite this article:** Makonin, S. *et al.* Electricity, water, and natural gas consumption of a residential house in Canada from 2012 to 2014. *Sci. Data* 3:160037 doi: 10.1038/sdata.2016.37 (2016).

## Supplementary Material



## Figures and Tables

**Table 1:** House Windows.

			**Window Size (cm)**			
**Location**	**Sub-Location**	**Direction**	**Width**	**Height**	**Window Type**	**Notes**
Main Floor	Bathroom	North	63.818	97.155	A/O	obscure, top vent open outward
Main Floor	Bedroom	South	121.603	117.475	XO	50% sliding vents
Main Floor	Bedroom	North	152.400	101.918	XO	50% sliding vents
Main Floor	Dining Room	North	184.785	132.398	XOX	18” sliding vents
Main Floor	Master Ensuite	East	63.818	97.155	A/O	obscure, top vent open outward
Main Floor	Kitchen	North	89.218	83.820	XO	50% sliding vents
Main Floor	Master Bedroom	South	200.025	101.918	XOX	18” sliding vents
Main Floor	Stairwell/Kitchen	North	150.178	117.475	XO	50% sliding vents
Main Floor	Living Room	South	306.705	132.398	XOX	24” sliding vents
Basement	Rec Room	East	73.978	99.378	OX	50% sliding vents, laminated
Basement	Home Office	East	199.708	99.060	XOX	18” sliding vents, laminated
Rental Suite	Bedroom	North	73.978	89.218	OX	50% sliding vents, laminated
Rental Suite	Kitchen	West	73.660	73.660	OX	50% sliding vents, laminated
Rental Suite	Living Room	West	73.660	73.660	OX	50% sliding vents, laminated
House’s Total Window Surface (m^2^)				19.738		

**Table 2:** Metering Equipment Specifications.

**Meter**	**Resource**	**Make**	**Model**	**Manuals/Specs (.pdf)**	**Sample Rate**	**Accuracy Standards**	**Measurement Technology**
WHE (incl. all sub-meters)	electricity	DENT	PowerScout 18	Metering_DENTps18	1 Hz sampling	1% (<0.5% typical)ANSI C12.20IEC 62053	digital DSP
WHW (v1)	water	DJL Meter	DLJSJ75C	Metering_DLJSJ75C	pulse/Gallon	AWWA C712ISO 4046 Class C	single-jet (inferential) impeller
HTW (v1)	water	Elster	S130	Metering_S130hot	pulse/Gallon	unknown	single-jet (inferential) impeller
WHW (v2)HTW (v2)	water	Elster/Kent	V100	Metering_KentV100	pulse/0.5 l	UK WRc BS5728ISO 4064 Class C/D	volumetric, grooved piston
WHG	natural gas	Elster	AC250	Metering_AC-250	pulse/25dm^3^	ANSI B109.1Measurement Canada	exterior temperature compensatedgas diaphragm
FRG	natural gas	Elster	BK-G4	Metering_BK-G4	pulse/1 ft^3^	EN 1359	interior gas diaphragm
Modbus	data	Obvius	EMB-A8810	Metering_EMB-A8810	read/minute		
Pulse	data	Obvius	EMB-A7810	Metering_EMB-A7810	read/minute		

**Table 3:** File Descriptions.

**File Name**	**Description**
Climate_HistoricalNormals.csv	A summary of climate normals observed from the years between 1981 and 2010 measured from Environment Canada’s weather station at Burnaby Mountain (which closed in 2010).
Climate_HourlyWeather.csv	Hourly weather data measured from Environment Canada's weather station at YVR (Vancouver International Airport).
Electricity_???.csv	There is a file of electricity measurements for each meter and sub-meter: B1E, B2E, BME, CDE, CWE, DNE, DWE, EBE, EQE, FGE, FRE, GRE, HPE, HTE, OFE, OUE,RSE, TVE, UTE, WHE, and WOE (see Table 2).
Electricity_?.csv	There is a file for each instantaneous measurement (I, P, Q, and S) that has each meter, sub-meter, and soft-meter as columns. These files are convenient for training and testing disaggregators and eco-visualizations. There is no need to parse a specific column value for each of the sub-meter files which can be time consuming.
Electricity_Billing.csv	Data values collected from each power bill statement.
Electricity_Monthly.csv	Monthly consumption data downloaded from utility used to create Fig. 4.
Electricity_Statements.pdf	Redacted copies of each of the power bill statements received during the data collection period.
Manual_*.pdf	Manuals for appliances listed in Table 3.
Metering_*.pdf	Technical specifications and documentation of the metering equipment used.
NaturalGas_Billing.csv	Data values collected from each gas bill statement.
NaturalGas_FRG.csv	Natural gas consumption measurements from the furnace gas sub-meter.
NaturalGas_HeatValues.csv	The daily measured heat values downloaded from utility. Measurements in GJ/103 m^3^. Our test house is in Zone 24 (Lower Mainland).
NaturalGas_Monthly.csv	Monthly consumption data downloaded from utility used to create Fig. 4.
NaturalGas_Statements.pdf	Redacted copies of each of the gas bill statements received during the data collection period.
NaturalGas_WHG.csv	Natural gas consumption measurements from the whole-house gas meter.
Water_Billing.csv	Data values collected from each City of Burnaby annual utility bill statement.
Water_DWW.csv	Water consumption measurements for the dishwasher. Annotated by hand.
Water_HTW.csv	Water consumption measurements from the instant hot water unit sub-meter.
Water_QualityReport_2012.pdf	The City of Burnaby annual water quality report for 2012.
Water_QualityReport_2013.pdf	The City of Burnaby annual water quality report for 2013.
Water_QualityReport_2014.pdf	The City of Burnaby annual water quality report for 2014.
Water_WHW.csv	Water consumption measurements from the whole-house water meter.
Water_ZonesMap.pdf	Map of the City of Burnaby shown the different water zones. Our test house is in Zone 585 (North Burnaby).

**Table 4:** Column Name/Field Descriptions

**Field Name**	**Description**
unix_ts	The unix timestamp integer value of a given reading. The house used in AMPds is in the America/Vancouver timezone. So the timestamp 1333263600 is equivalent to April 01, 2012 12:00:00 AM (local time) or Apr 01, 2012 07:00:00 (GMT or UTC). Timestamps having a plus sign in front of it (e.g., +1333263600) denotes that that reading had missing data and the record was created algorithmically.
V	Line voltage measured in volts to the nearest deci-volt. Typically 120 V for single line and 240 V for double line.
I	Line current measured in amperes to the nearest deci-amperes.
f	Line frequency, in hertz (Hz) accurate to two decimal places. Typically 60 Hz.
DPF	Displacement power factor or just power factor (PF), the cosine of the voltage and current phase angle. It measures how out-of-phase voltage and current are. Has values ranging from −1 to 1 with two decimal places of accuracy.
APF	Apparent power factor, the ratio of real power over apparent power with harmonics included. Has values ranging from −1 to 1 with two decimal places of accuracy.
P	Real power, an instantaneous integer value measured in VAR.
Pt	Real energy, a cumulative integer value measured in VARh.
Q	Reactive power, an instantaneous integer value measured in VAR.
Qt	Reactive energy, a cumulative integer value measured in VARh.
S	Apparent power, an instantaneous integer value measured in VA.
St	Apparent energy, a cumulative integer value measured in VAh. This is the measurement that power utility companies bill for and appears as kWh on a power bill.
counter	A cumulative decimal value that increases with every pulse of the water or natural gas meter. At each pulse the counter increases the amount of the pulse measurement value. For example, a water meter which pulses every 0.5 l would have its count increase 0.5 every pulse. Water meters are measured in litres (l) and gas meters are measured in cubic decimeters (dm^3^).
avg_rate	The average rate is the difference of the current reading and the previous reading multiplied by the measure rate. For example, per minute readings are multiplied by 1 and per hour readings are multiplied by 60. Water meters are measured in litres per minute (l/min) and gas meters are cubic deci-meters per hour (dm^3^/h).
inst_rate	The instantaneous rate is calculated based on the time it takes for a certain number of pulses to be received. For example, if it took 50 s to receive five pulses, the rate would be 5 [pulses]/50 [seconds]=0.1 [pulses/second]. Water meters are measured in litres per minute (l/min) and gas meters are cubic deci-meters per hour (dm^3^/h).
WHE	The whole-house power meter which is equivalent to the power utility’s meter. Line voltage is 240 V and has a 200A double-pole breaker.
RSE	The basement rental suite sub-meter which has a line voltage of 240V and has a 100A double-pole breaker.
GRE	The detached garage sub-meter which has a line voltage of 240 V and has a 60A double-pole breaker.
MHE	The main house soft-meter which is calculated by MHE=WHE—(RSE+GRE).
B1E	Sub-meter for the north bedroom plugs and lights which has a line voltage of 120V and has a 15A single-pole breaker.
B2E	Sub-meter for the master and south bedroom plugs and lights which has a line voltage of 120 V and has a 15A single-pole breaker.
BME	Sub-meter for the some of the basement plugs and lights which has a line voltage of 120 V and has a 15A single-pole breaker. A small freezer is powered by this breaker.
CWE	Sub-meter for the front loading clothes washer which has a line voltage of 120 V and has a 15A single-pole breaker.
DWE	Sub-meter for the kitchen dishwasher which has a line voltage of 120 V and has a 15A single-pole breaker.
EQE	Sub-meter for the security and network equipment which has a line voltage of 120 V and has a 15A single-pole breaker.
FRE	Sub-meter for the forced air furnace fan and thermostat which has a line voltage of 120 V and has a 15A single-pole breaker.
HPE	Sub-meter for the heat pump which has a line voltage of 240 V and has a 40A double-pole breaker.
OFE	Sub-meter for the home office lights and plugs which has a line voltage of 120 V and has a 15A single-pole breaker.
UTE	Sub-meter for the utility room plug which has a line voltage of 120 V and has a 15A single-pole breaker.
WOE	Sub-meter for the kitchen convection wall oven which has a line voltage of 240 V and has a 30A double-pole breaker.
CDE	Sub-meter for the clothes dryer which has a line voltage of 240 V and has a 30A double-pole breaker.
DNE	Sub-meter for the dining room plugs which has a line voltage of 120 V and has a 15A single-pole breaker.
EBE	Sub-meter for the electronics workbench which has a line voltage of 120 V and has a 15A single-pole breaker.
FGE	Sub-meter for the kitchen fridge which has a line voltage of 120 V and has a 15A single-pole breaker.
HTE	Sub-meter for the instant hot water unit which has a line voltage of 120 V and has a 15A single-pole breaker.
OUE	Sub-meter for the outside plug which has a line voltage of 120 V and has a 15A single-pole breaker.
TVE	Sub-meter for the entertainment equipment (TV, PVR, amplifier, and Blu-Ray) which has a line voltage of 120 V and has a 15A single-pole breaker.
UNE	The unmetered soft-meter amount with is calculated by UNE=MHE—sum(all sub-meters under MHE).

**Table 5:** Major House Appliance Details

**Appliance**	**Meter IDs**	**Location**	**Sub-Location**	**Make**	**Model**	**Manuals/Specs (.pdf)**	**EnergyGuide/EnerGuide Rating**	**Notes/Service History**
Clothes Dryer	CDE	Basement	Hallway	Frigidaire/Electrolux	FEQ1442CES0	Manual_FEQ1442	938 kWh/year	
Clothes Washer	CWE, CWW	Basement	Hallway	Frigidaire/Electrolux	FTF2140ES0	Manual_FTF2140	247 kWh/year	
Dishwasher	DWE, DWW	Main Floor	Kitchen	Kenmore/ Sears Roebuck	587.144034	Manual_587144034	300 kWh/year	setup to only use hot water, cold water not connected
Fridge	FGE	Main Floor	Kitchen	Amana/Maytag	ABB2227DES	Manual_Amana	488 kWh/year	capacity 21.9 Cu.Ft., cooling setting 3, freezer setting 4
Fireplace	FPG	Main Floor	Living Room	Napoleon	STARfire GD70NT-S	Manual_StarFire		rarely used
Thermostat (v1)	FRE	Main Floor	Hallway	Proliphix	IM550w	Manual_IMT550 Manual_PTS-OS3		PTS-OS3 is the out door temperature sensor Oct 31, 2012: replaced with Nest.
Thermostat (v2)	FRE	Main Floor	Hallway	Nest Labs	Thermostat Gen 2	Manual_Nest		outdoor temperature retrieve from weather service website
HVAC Furance & Fan	FRE, FRG	Basement	Furnace Room	American Standard	Freedom 90 Comfort-R AUY060R9V3W5	Manual_18CD20D3 Manual_X341119P12	93% AFUE	Stage 1: 39,000 BTUH, Stage 2: 60,000 BTUH
Garage Door	GRE (part of)	Garage		Stanley	440.51			1/3 HP, 120 V, 7 A Max, 14×6.6 ft door
Heat Pump	HPE	Outside		American Standard	Heritage 16 4A6H6036B1000AA	Manual_Heritage16 Manual_4a6h6036 Manual_TXCSpec	SEER 17 HSPF 9.2	2-stage compressor
Hot Water Unit	HTE, HTW, HTG	Basement	Hallway	Rinnai	R53i-0 (REU-V2520FFU-US)	Manual_R53i_op Manual_R53i_service Manual_R53i_spec	187 Therms/year	3/4” gas in-pipe, 3/4” water in- and out-pipe, water meter installed on in-pipe Jul 14, 2012: serviced for internal leaks.
Stovetop	STG	Main Floor	Kitchen	Bosch	PGL985UC/01	Manual_pgl985uc		44800 BTU/hr (13.12 kW)
Amp	TVE (part of)	Basement	Rec Room	Marantz	NR1403	Manual_NR1403		
Blu-Ray Player	TVE (part of)	Basement	Rec Room	Panasonic	DMP-BDT210	Manual_DMPBDT2		
DVR/PVR (portal)	TVE (part of)	Basement	Rec Room	Arris	Portal MP2000NA	Manual_Arris		115 VAC, 0.3 A Max
Sub-Woofer	TVE (part of)	Basement	Rec Room	Definitive	PowerField SuperCude II	Manual_SuperCubes		
Television	TVE (part of)	Basement	Rec Room	Panasonic	TC-P50ST30	Manual_TCP42ST30	191 kWh/year	
Furnace UV Filter	UTE	Basement	Furnace Room	Sanuvox	R4000GX	Manual_Sanuvox		
Wall Oven	WOE	Main Floor	Kitchen	Bosch	HBL5045AUC/01	Manual_hbl5045auc		0.14/3.5 kW, CSA 289 kWh
Bathroom Vent	MHE (part of)	Main Floor	Bathroom	Broan	QTR090C	Manual_QTR090C	Energy Star	108 W of power, 90 CFM, 1.0 Sones
Bathroom Vent	MHE (part of)	Basement	Bathroom	Broan	QTR090C	Manual_QTR090C	Energy Star	108 W of power, 90 CFM, 1.0 Sones
Chest Freezer	MHE (part of)	Basement	Hallway	Igloo	FRF434	Manula_Igloo	212 kWh/year	capacity 3.6 Cu.Ft., manual defrost, cooling setting 3
Cooking Vent	MHE (part of)	Main Floor	Kitchen	Faber	Diamante Pro 36” 630001088	Manual_Faber		4.5A/530W, 3×20 W halogen lamps max, 600 CFM, 6.0 Sones
DVR/PVR (main)	MHE (part of)	Basement	Home Office	Arris	Gateway MG5522G/NA	Manual_Arris		115 VAC, 1.5 A Max
Hot Water Kettle	MHE (part of)	Main Floor	Kitchen	Cuisinart	CJK-17BC	Manual_cjk17bc		1.7 l capacity, 1500W of power for quick heating, using in the morning and other times
Mini-Oven	MHE (part of)	Main Floor	Kitchen	Breville	B0V800XL/A	Manual_Breville		rated at using 1800 W of power
Rental - Fridge	RSE (part of)	Rental Suite	Kitchen	Maytag	Performa PTB1952GRW	Manual_Performa	489 kWh/year	18.5 cu. ft. capacity
Rental - Stove/Oven	RSE (part of)	Rental Suite	Kitchen	Frigidaire/Electrolux	CMEF212ES	Manual_CMEF212ES		2.9 Cu. Ft., 24" Electric, Manual Clean, 1×6" 1250 w Element, 2×6' 1500 w Coil Elements, 1×8" 2600 w Coil Element, Lift-up Cooktop, 4 Pass Bake Element 2400 w, 6 Pass Broil 2750 w Element, 1 Oven Light, 1 Appliance Outlet
Rental - Bathroom Vent	RSE (part of)	Rental Suite	Bathroom	Broan	QTR090C	Manual_QTR090C	Energy Star	108 W of power, 90 CFM, 1.0 Sones
Rental - Clothes Dryer	RSE (part of)	Rental Suite	Bedroom	Samsung	DV4006JW2	Manual_DV4006		
Rental - Clothes Waher	RSE (part of)	Rental Suite	Bedroom	Samsung	P801DWN/XAC	Manual_P801		
Rental - Cooking Vent	RSE (part of)	Rental Suite	Kitchen	Cypress	SB3030	Manual_AC8830		2×100 W, 900 CFM, lighting:40 W
